# Acute activation of human epithelial sodium channel (ENaC) by serum and glucocorticoid inducible kinase 1 (SGK1) requires prior cleavage of the channel’s γ-subunit at its proximal cleavage site

**DOI:** 10.1007/s00424-025-03099-z

**Published:** 2025-06-21

**Authors:** Alexei Diakov, Florian Sure, Alexandr V. Ilyaskin, Christoph Korbmacher

**Affiliations:** Institute of Cellular and Molecular Physiology, Friedrich-Alexander-Universität Erlangen-Nürnberg, Waldstr. 6, 91054 Erlangen, DE Germany

**Keywords:** Epithelial sodium channel (ENaC), Serum and glucocorticoid inducible kinase 1 (SGK1), Proteolytic ENaC activation, *Xenopus laevis* oocytes, Patch clamp

## Abstract

Serum and glucocorticoid inducible kinase 1 (SGK1) is a key regulator of the epithelial sodium channel (ENaC). In rat ENaC, the serine residue 621 (S621) in the channel’s α-subunit is essential for acute channel activation by SGK1 in outside-out patches. Phosphorylation at S621 probably turns previously silent channels into channels with a high open probability. This is reminiscent of proteolytic ENaC activation resulting from cleavage of the channel’s γ-subunit at specific proximal and distal cleavage sites and the release of an inhibitory peptide tract. The first aim of this study was to demonstrate that human ENaC could also be activated acutely by SGK1 and that this depended on the homologous phosphorylation site S594 in human αENaC. Secondly, we wanted to explore whether human ENaC activation by SGK1 depended on the cleavage state of γENaC. Outside-out patch-clamp recordings in *Xenopus laevis* oocytes expressing human αβγENaC revealed the critical importance of S594 for acute channel activation by SGK1. The latter was not additive to proteolytic channel activation. Interestingly, preventing proximal cleavage in human γENaC completely abolished the stimulatory effect of SGK1. Moreover, tethering the inhibitory peptide in γENaC to its binding site via an engineered disulfide bond prevented stimulation by SGK1. We conclude that ENaC activation by SGK1 requires prior cleavage of γENaC at its proximal cleavage site. Together, these results reveal that SGK1-mediated stimulation of human ENaC is intricately linked to the proteolytic processing of the channel’s γ-subunit, emphasizing a previously underappreciated interplay between kinase and protease regulatory pathways.

**Supplementary Information:**

The online version contains supplementary material available at 10.1007/s00424-025-03099-z.

## Introduction

The epithelial sodium channel (ENaC) is a member of the ENaC/degenerin ion channel family and serves as the rate-limiting step for Na^+^ absorption in several epithelial tissues. These include the distal nephron, where ENaC plays a crucial role in regulating body Na^+^ homeostasis, extracellular fluid volume, and long-term blood pressure [19, 29, 42, 54].

ENaC is a heterotrimeric ion channel, typically consisting of three homologous subunits (α, β, and γ) [46, 47]. In humans, but not in rodents, the α-subunit can be replaced by a δ-subunit, altering channel function [21, 69, 71, 72]. Each subunit contains two transmembrane domains, a large extracellular loop, and short cytosolic N- and C-termini. ENaC activity is tightly regulated by several hormonal and local mediators [28, 32, 39, 49, 56]. Among these, the mineralocorticoid aldosterone is believed to be the key hormonal regulator in the distal nephron. Upon binding to the mineralocorticoid receptor, aldosterone activates intracellular signaling pathways that ultimately increase ENaC activity at the cell surface.

One of the critical mediators in this process is the serum and glucocorticoid-inducible kinase 1 (SGK1). Transcription of SGK1 is enhanced in response to aldosterone [7, 40, 43, 58, 62] and leads to an increase in ENaC abundance at the cell surface. There is good evidence that SGK1 achieves this—at least in part—by hampering ENaC’s interaction with the ubiquitin ligase Nedd4-2, which otherwise marks ENaC for internalization and proteosomal degradation [56, 59–61]. SGK1 also promotes forward trafficking of ENaC to the plasma membrane [1, 37, 38]. In the *Xenopus laevis* oocyte expression system, both mechanisms are likely to contribute to the stimulatory effect of co-expressed SGK1 on ENaC expression at the cell surface [1, 8, 12, 33, 52].

In addition, we demonstrated that constitutively active human SGK1 acutely enhanced rat ENaC activity in excised outside-out patch-clamp recordings [10]. This finding provided direct evidence that, beyond its role in regulating ENaC abundance at the cell surface, SGK1 also increased the channel’s open probability. Importantly, this stimulatory effect of SGK1 depended on the serine residue S621, which is part of a phosphorylation site located in the intracellular C-terminal domain of rat αENaC [10, 12]. In a recent study, we provided evidence that SGK1 may not directly phosphorylate this critical serine residue but may increase the activity of proline-directed kinases, such as dual specificity tyrosine-phosphorylation-regulated kinase 2 (DYRK2), to phosphorylate this site [11]. Indeed, it is likely that ENaC regulation by this stimulatory phosphorylation site and an adjacent inhibitory phosphorylation site (S617 in rat) involves a complex interplay of several kinases and phosphatases [10–12]. In any case, it is clear from these latter studies that in outside-out patches the acute stimulatory effect of SGK1 on rat ENaC critically depends on the S621 phosphorylation site. It remains to be shown that SGK1 can also activate human ENaC in a similar manner and that the homologous phosphorylation site in human αENaC (S594) plays a critical role in mediating this stimulatory effect.

In addition to its regulation by kinases, ENaC activity is also controlled by a unique mechanism known as proteolytic channel activation [25, 31, 55, 65]. This mechanism involves proteases activating the channel by cleaving specific inhibitory peptide domains from the α- and γ-subunits [3, 6, 48]. According to the current paradigm, intracellular proteases such as furin and related convertases perform both required cleavage events in αENaC, while they only complete the proximal cleavage event in γENaC. These cleavage events are thought to occur already intracellularly along the biosynthetic pathway [24, 25]. In contrast, the pivotal final cleavage event at the distal cleavage site of γENaC leading to full channel activation is believed to occur at the cell surface [9, 20]. The proteases involved in this critical event are still incompletely understood [28, 31]. Recently, transmembrane serine protease 2 (TMPRSS2) has been identified as a key player in proteolytic ENaC activation in human respiratory epithelial cells and in mouse kidney [62, 63].

Interestingly, in outside-out patch-clamp recordings with single-channel resolution proteolytic ENaC activation by extracellular application of the prototypical serine proteases trypsin or chymotrypsin closely resembles the acute stimulatory effect of constitutively active SGK1 included in the pipette solution. In both cases, ENaC activity is enhanced through an increase in single-channel open probability and the recruitment of previously near-silent channels, leading to greater ENaC-mediated currents [4, 9, 10, 20]. The similarity of protease- and kinase-mediated ENaC activation suggests a functional interplay of the underlying molecular mechanisms.

The aim of the present study was twofold: First, we wanted to demonstrate that human ENaC could be acutely stimulated by SGK1 and that this stimulatory effect depended on S594 homologous to S621 in rat αENaC. Second, we wanted to investigate whether SGK1-mediated stimulation of ENaC depended on the cleavage state of its γ-subunit.

## Materials and methods

### Plasmids

Full-length complementary DNAs (cDNAs) encoding human wild-type α-, β-, and γENaC were originally obtained from Harry Cuppens [2, 23, 51]. cDNA encoding the short isoform of human wild-type δENaC was provided by Rainer Waldmann (Valbonne, France) [69]. cDNAs were subcloned into the pGEM-HE vector for heterologous expression in *Xenopus laevis* oocytes. Plasmids were linearized using Mlu1 restriction enzymes (Mlu1-HF, New England Biolabs) and used as templates for cRNA synthesis using SP6 RNA polymerase (mMessage mMachine, Ambion). QuickChange Lightning site-directed mutagenesis kit (Agilent Technologies) was used to introduce a S594A mutation in αENaC, 135RKRK138AAAA, S155C, and Q426C mutations in γENaC. The same kit was used to introduce a V5-tag (GKPIPNPLLGLDST) between amino acids 1 and 2 in the N-terminus of γENaC for experiments shown in Fig. 3 and to generate the chimeric δ-α-subunit used for experiments shown in Supplementary Fig. 1b. Sequences were routinely confirmed by sequence analysis.

### Isolation of *Xenopuslaevis* oocytes and injection of cRNA

Oocytes from *Xenopus laevis* were isolated essentially as described previously [10, 12, 26, 62–64]. Ovarian lobes were excised by partial ovariectomy under anesthesia with Tricain 0.2%, in accordance with the principles of German legislation, with approval by the animal welfare officer of the University of Erlangen-Nürnberg (FAU), and under the governance of the state veterinary health inspectorate. Oocytes were isolated from ovarian lobes using a type-2 collagenase from *Clostridium histolyticum* (Sigma-Aldrich). Defolliculated stage V–VI oocytes were injected with cRNA using 0.1–0.5 ng of cRNA per ENaC subunit (α or δ, β, and γ) per oocyte. To prevent Na^+^ overloading, injected oocytes were incubated in low sodium solution where NaCl (95 mM) was replaced by N-methyl-D-glucamin [NMDG]-Cl (in mM: 95 NMDG-Cl, 1 NaCl, 4 KCl, 1 CaCl_2_, 1 MgCl_2_, 10 HEPES [4-(2-hydroxyethyl)−1-piperazineethanesulfonic acid], 7.4 pH adjusted with Tris(hydroxymethyl)aminomethan [Tris]), supplemented with 100 U/ml sodium penicillin and 100 µg/ml streptomycin sulfate. Unless stated otherwise, oocytes were studied 48–72 h after injection.

### Two-electrode voltage-clamp experiments in *Xenopus* *laevis* oocytes

Two-electrode voltage-clamp measurements were performed essentially as described previously [9, 10, 26, 62–64]. Bath solution exchanges with a gravity-fed system were controlled by a magnetic valve system (ALA BPS-8; ALA Scientific Instruments). Oocytes were clamped at a holding potential of − 60 mV using an OC-725C amplifier (Warner Instruments) connected by an LIH-1600 (HEKA) to a personal computer. Pulse 8.78 software (HEKA) was used for data acquisition. ND96 was used as a standard bath solution (composition in mM: 96 NaCl, 2 KCl, 1.8 CaCl_2_, 1 MgCl_2_, 5 HEPES; pH 7.4 adjusted with Tris). Amiloride-sensitive current (∆*I*_Ami_) was determined by subtracting the current value recorded in the presence of amiloride (2 μM) from the current value in its absence.

### Recordings in outside-out macropatches excised from *Xenopus laevis* oocytes

Current recordings from outside-out membrane patches were performed as described previously [10–12, 33] using conventional patch-clamp technique. Patch pipettes were pulled from borosilicate glass capillaries and had a tip diameter of about 5–7 μm after fire polishing. Pipettes were filled with K-gluconate pipette solution (in mM: 90 K-gluconate, 5 NaCl, 2 Mg-ATP, 2 EGTA, 10 HEPES; pH 7.28 adjusted with Tris). Seals were routinely formed in a low sodium NMDG-Cl bath solution (in mM: 95 NMDG-Cl, 1 NaCl, 4 KCl, 1 MgCl_2_, 1 CaCl_2_, 10 HEPES; pH 7.4 adjusted with Tris). In this bath solution, the pipette resistance averaged about 3 MΩ. In NaCl bath solution, NMDG-Cl was replaced by 95 mM NaCl. For continuous current recordings, the holding potential was set to − 70 mV using an EPC9 amplifier (HEKA Elektronik, Lambrecht, Germany). Using a 3 M KCl flowing boundary electrode, the liquid junction (LJ) potential occurring at the pipette/NaCl bath junction was measured to be 12 mV (bath positive) [35]. Thus, at a holding potential of − 70 mV, the effective trans-patch potential was − 82 mV. This value is close to the calculated equilibrium potential of Cl^−^ (*E*_Cl−_ = − 77.4 mV) and K^+^ (*E*_K+_ = − 79.4 mV) under our experimental conditions. Experiments were performed at room temperature. To change from one bath solution to another, a conventional gravity-fed system controlled by a magnetic valve system (ALA BPS-8) was used in combination with a TIB14 interface (HEKA Elektronik, Lambrecht, Germany). Pulse 8.78 software (HEKA Elektronik, Lambrecht, Germany) was used for data acquisition. Like in two-electrode voltage-clamp experiments (see above), the amiloride-sensitive current (∆*I*_Ami_) in outside-out membrane patches was determined by subtracting the current value recorded in the presence of 2 μM amiloride (100 µM for experiments in Supplementary Fig. 1) from the corresponding value recorded prior to its addition. The current traces were filtered at 200 Hz and sampled at 800 Hz.

### Cell surface protein detection and western blot analysis in *Xenopus laevis* oocytes

To separate cell surface proteins from intracellular proteins, a biotinylation approach was used, essentially as described previously [9, 26, 34, 62, 63]. Briefly, cell surface proteins were marked by a 30-min incubation of oocytes in 1 mg/ml EZ-linked sulfo-NHS-SS-Biotin (Sigma-Aldrich), before quenching remaining biotin with glycine and subsequent cell lysis. Thereafter, biotinylated proteins were extracted by overnight incubation with Neutravidin beads (Pearce). Protein samples were boiled for 5 min at 95 °C and subjected to 10% SDS-PAGE and western blot analysis.

C-terminal cleavage fragments of human γENaC were detected using a subunit-specific antibody generated in rabbits against human γENaC (Pineda Antibody Service) [21, 26] at a dilution of 1:5000 and horseradish peroxidase-labeled secondary goat anti-rabbit antibodies (Santa Cruz Biotechnology) at a dilution of 1:50,000. N-terminal cleavage fragments of the V5-tagged γENaC were detected using mouse monoclonal anti-V5 antibody (Invitrogen) at a dilution of 1:1000 and a secondary horseradish peroxidase-labeled goat anti-mouse antibody (Abcam) at a dilution of 1:50,000. To validate separation of cell surface proteins from intracellular proteins by biotinylation, blots stained with the anti-V5 antibody were stripped and reprobed using a polyclonal rabbit anti-β-actin antiserum (Sigma-Aldrich) at a dilution of 1:5000.

### Chemicals

Recombinant constitutively active human SGK1 (∆1–60, S422D) was purchased from Biomol GmbH (Hamburg, Germany) as 2 μg (SGK1) vials in 50 μl stock solution containing as main components 50 mM Tris–HCl, 0.1 mM EGTA, 0.1% 2-mercaptoethanol, 0.15 mM NaCl, and 270 mM sucrose. SGK1 pipette solutions were freshly prepared on the day of the experiment by adding an appropriate amount of SGK1 stock solution to 1 ml of the corresponding pipette solution, giving a final SGK1 concentration of 80 U/ml. To preserve SGK1 activity, the pipette solution was supplemented with dithiothreitol (Sigma-Aldrich) in a concentration of 0.1 mM. Control experiments were performed using heat-inactivated SGK1, which had been incubated at 68 °C for 45 min. Amiloride hydrochloride and trypsin from bovine pancreas were both purchased from Sigma-Aldrich and were added from aqueous 10 mM or 200 µg/ml stock solutions, respectively, to achieve final concentrations of 2–100 µM or 2 µg/ml, as indicated in the figure legends. S3969 was synthesized essentially as described previously [23, 41, 64]. A 10 µM S3969 bath solution was prepared from a 100 mM stock solution in DMSO (dimethylsulfoxide).

### Statistical methods

Electrophysiological recordings were analyzed using the software Nest-o-patch (https://sourceforge.net/projects/nestopatch/) developed by Dr. Viatcheslav Nesterov (Institute of Cellular and Molecular Physiology, Friedrich-Alexander-Universität Erlangen-Nürnberg, Erlangen, Germany). Data are presented as mean values ± SEM. *n* indicates the number of individual experiments; *N* indicates the number of different batches of oocytes used. Normal distribution of data was assessed using the D′Agostino-Pearson omnibus or Shapiro–Wilk test. Statistical significance was assessed by an appropriate parametric test: paired or unpaired Student’s *t* test or repeated-measures analysis of variance (ANOVA) with Dunnett’s post-hoc test. To assess normalized effects, values were log-transformed before statistical testing. Significance was accepted for *p* < 0.05. *, **, and *** represent *p* values smaller than 0.05, 0.01, and 0.001, respectively. Statistical analysis was performed using Graph Pad Prism 5.04 and R environment for statistical computing v. 4.1 (R Core team).

## Results

### Recombinant SGK1 stimulates human ENaC in outside-out patches from *Xenopuslaevis* oocytes

To investigate whether SGK1 acutely stimulates the activity of human ENaC, we performed outside-out patch-clamp recordings in *Xenopus laevis* oocytes expressing α-, β-, and γ-subunits of human ENaC (αβγENaC). Recordings from outside-out macropatches were started approximately 4 min after patch excision. To minimize spontaneous channel rundown, known to occur in the presence of a high extracellular Na^+^ concentration [68], baseline current recordings were performed in a NMDG-Cl bath solution containing only 1 mM Na^+^. Under these conditions, inward currents were negligible at a holding potential of − 70 mV. In contrast, periodic exposure to a 95 mM NaCl bath solution revealed sizeable inward currents, consistent with a current component carried by Na^+^ influx via ENaC. Indeed, this current component was largely inhibited by the application of 2 μM amiloride, a concentration known to inhibit αβγENaC almost completely [30, 36, 41]. ENaC activity was monitored over time by repeatedly determining the amiloride-sensitive sodium current (∆*I*_Ami_).

**Fig. 1:**
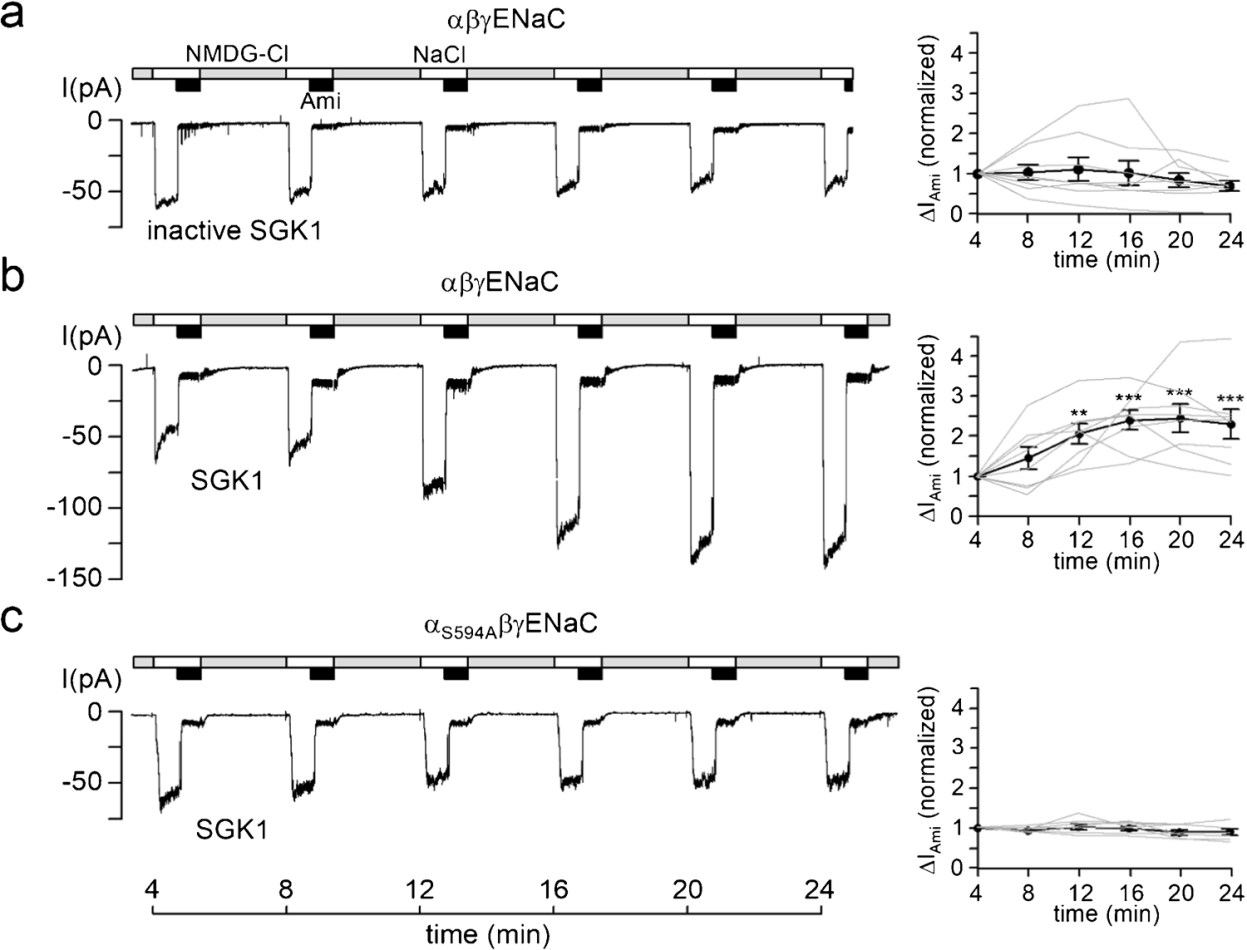
Recombinant SGK1 stimulates human ENaC currents in outside-out patches from *Xenopus laevis* oocytes and depends on a conserved serine residue in αENaC.** a, b, c** *Left panels:* Representative current traces recorded in outside-out macropatches of human αβγENaC (**a**, **b**) or α_S594A_βγENaC (**c**) expressing oocytes at a holding potential of − 70 mV. As indicated by the bars, bath solution was changed from a low Na^+^ (*grey bars*; NMDG-Cl; [Na^+^] = 1 mM) to a normal Na^+^ containing solution (*white bars*; NaCl; [Na^+^] = 96 mM) without or with amiloride (*black bars*; Ami, 2 µM). Heat-inactivated (inactive SGK1; **a**) or active recombinant SGK1 (SGK1; 80 U/ml; **b**, **c**) were included in the pipette solution as indicated under the traces. *Right panels:* Summary of normalized Δ*I*_Ami_ values obtained from similar experiments as shown in the corresponding *left panels*. Absolute Δ*I*_Ami_ values were normalized to the initial Δ*I*_Ami_ value at *t* = 4 min in each recording. Each grey line corresponds to an individual outside-out patch clamp recording and connects Δ*I*_Ami_ values obtained at different time points. The black line in each graph connects average Δ*I*_Ami_ values (mean ± SEM). Δ*I*_Ami_ values at individual time points were compared with the corresponding initial Δ*I*_Ami_ value at 4 min using repeated-measures ANOVA with Dunnett's post-hoc test of log-transformed values. No statistical testing was performed to assess spontaneous current run-down (**a**, **c**). **p < 0.01, ***p < 0.001. **a** *n* = 8, *N* = 6; **b** *n* = 8, *N* = 7; **c** *n* = 7, *N* = 5

Figure 1a (left panel) shows a representative control recording with heat-inactivated SGK1 in the pipette solution. Summary data from similar experiments (Fig. 1a, right panel) indicate that under control conditions, ENaC currents in outside-out patches remain pretty stable over ~ 30 min, consistent with previous findings using vehicle controls in the pipette solution or other heat-inactivated kinases [10–12]. In contrast, when catalytically active SGK1 was included in the pipette solution, ∆*I*_Ami_ significantly increased by about twofold within ~ 25 min (Fig. 1b). This acute stimulatory effect of SGK1 on human ENaC is in good agreement with previous results from similar experiments with rat ENaC [10–12].

We have previously demonstrated that the serine residue S621 in the α-subunit of rat ENaC is essential for SGK1-mediated acute channel activation in outside-out patches [10–12]. To determine whether the homologous serine residue S594 in the α-subunit of human ENaC is similarly required, we performed outside-out patch-clamp recordings from oocytes expressing mutant α_S594A_βγENaC, in which S594 was replaced with alanine. Notably, SGK1 failed to stimulate α_S594A_βγENaC (Fig. 1c), indicating that S594 is critical for acute SGK1-mediated activation of human ENaC.

The human α-subunit can be replaced by the δ-subunit to form δβγENaC in the oocyte expression system. Therefore, we also tested the acute effect of SGK1 on δβγENaC in outside-out patch-clamp recordings, but observed no effect (Supplementary Fig. 1a). The absence of modulation by SGK1 may be due to a lack of a consensus motif for SGK1 in the C-terminal domain of δENaC. To test this, we generated a δ-α-chimeric subunit bearing the αENaC C-terminal phosphorylation site. However, SGK1 also failed to enhance the activity of ENaC in which the α-subunit was replaced by this δ-α-chimeric subunit (Supplementary Fig. 1b). Accordingly, all further experiments were confined to the αβγENaC configuration.

### Recombinant SGK1 fails to stimulate ENaC pre-treated with trypsin

At the single-channel level, acute SGK1-mediated ENaC activation [10] is reminiscent of proteolytic ENaC activation observed in single-channel recordings from outside-out patches exposed to the prototypical serine proteases trypsin or chymotrypsin [9, 20]. This similarity raises the question of whether these two mechanisms of channel activation are functionally linked and whether, following proteolytic channel activation, SGK1 can increase ENaC activity further.

To address this, we measured ∆*I*_Ami_ in outside-out macropatches excised from oocytes expressing αβγENaC, either with or without prior trypsin treatment (2 µg/ml, 3 min). In patches without trypsin pre-treatment, ∆*I*_Ami_ averaged 17.2 ± 6.2 pA (*n* = 4) at the first measurement ~ 4 min after patch excision. In contrast, patches pre-treated with trypsin exhibited significantly higher initial currents, averaging 88.6 ± 24.2 pA (*n* = 6; *p* < 0.05, Two-tailed unpaired Student’s *t*-test). The increased baseline currents indicate successful proteolytic activation of ENaC in patches from trypsin treated oocytes.

**Fig. 2:**
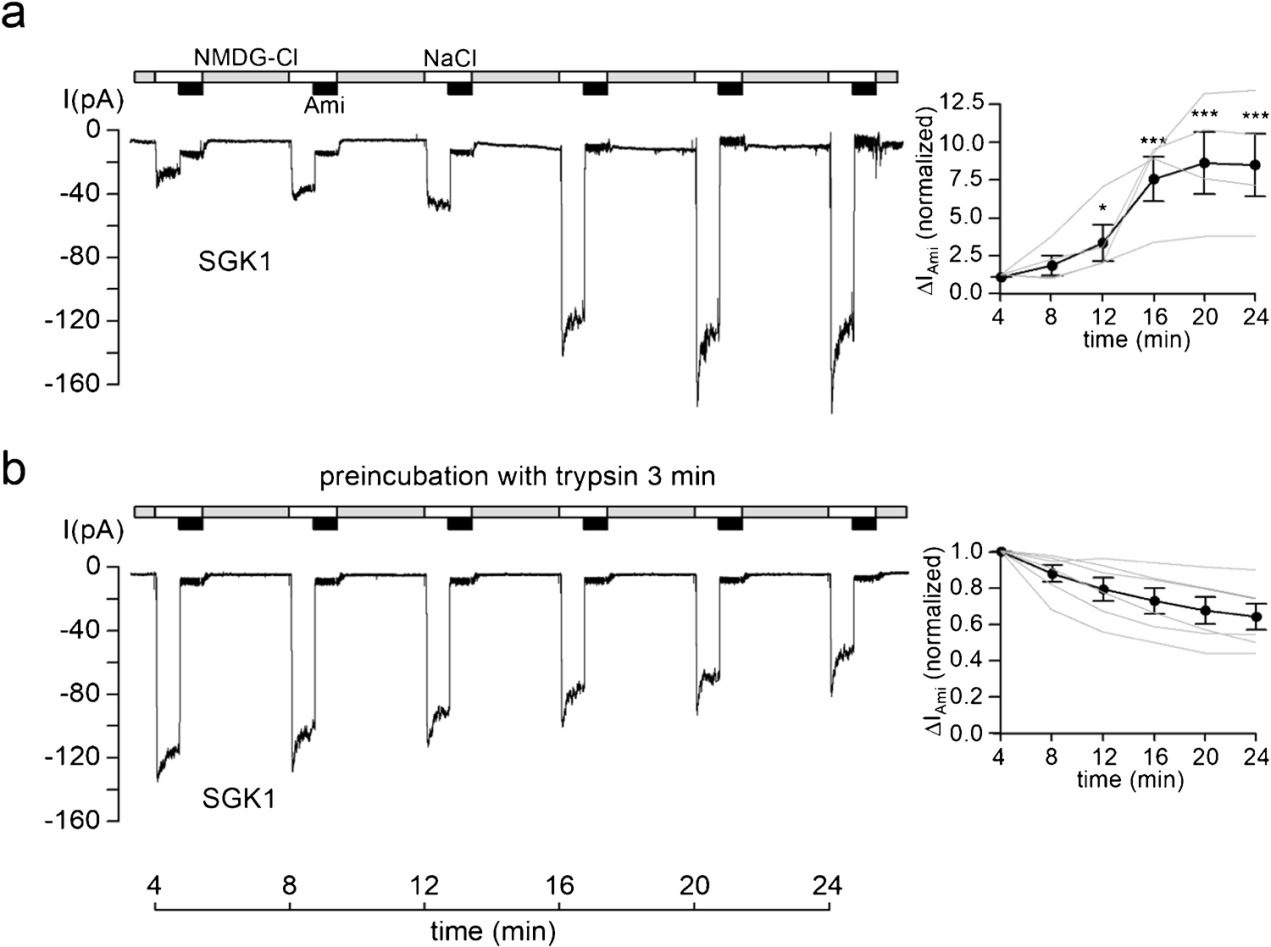
Recombinant SGK1 fails to stimulate ENaC currents in outside-out patches from oocytes pre-treated with the prototypical protease trypsin. **a, b** *Left panels*: Representative current traces recorded in outside-out macropatches of human αβγENaC as described in Fig. 1. Active recombinant SGK1 (SGK1; 80 U/ml) was included in the pipette solution as indicated below the traces. Before patch excision, oocytes were incubated in bath solution without (**a**) or with 2 µg/ml trypsin (**b**) for 3 min. *Right panels*: Summary of normalized Δ*I*_Ami_ values obtained from similar experiments as shown in the corresponding left panels using the same symbols as in Fig. 1. Δ*I*_Ami_ values at individual time points were compared with the corresponding initial Δ*I*_Ami_ value at 4 min using repeated-measures ANOVA with Dunnett's post-hoc test of log-transformed values. No statistical testing was performed for the traces shown in **b** with spontaneous current run-down. *p < 0.05, ***p < 0.001. **a** *n* = 4, *N* = 4; **b** *n* = 6, *N* = 4

Importantly, SGK1 failed to increase ∆*I*_Ami_ further in oocytes pre-treated with trypsin (Fig. 2b). In contrast, in matched control recordings without trypsin pre-treatment, SGK1 produced its characteristic stimulatory effect (Fig. 2a). The relative stimulatory effect of SGK1 in the control experiments without trypsin exposure was somewhat greater than the effect shown in Fig. 1a, possibly reflecting batch to batch variability of SGK1 activity or baseline ENaC open probability.

In summary, these findings suggest that the stimulatory effect of SGK1 on ENaC activity is not additive to proteolytic channel activation by trypsin.

#### Mutation of the proximal cleavage site in the channel’s γ-subunit prevents ENaC activation both by trypsin and by SGK1

In *Xenopus laevis* oocytes, γENaC undergoes proteolytic cleavage at the proximal but not the distal cleavage site before it reaches the cell surface. To determine whether proximal cleavage is a prerequisite for SGK1-mediated ENaC activation, we expressed wild-type α- and βENaC subunits with a γENaC mutant (γ_135AAAA138_) where the proximal cleavage site (^135^RKRK^138^) was replaced with alanines. This has been shown to prevent proximal cleavage of γENaC by furin or other endogenous convertases [3, 24, 53, 63].

**Fig. 3:**
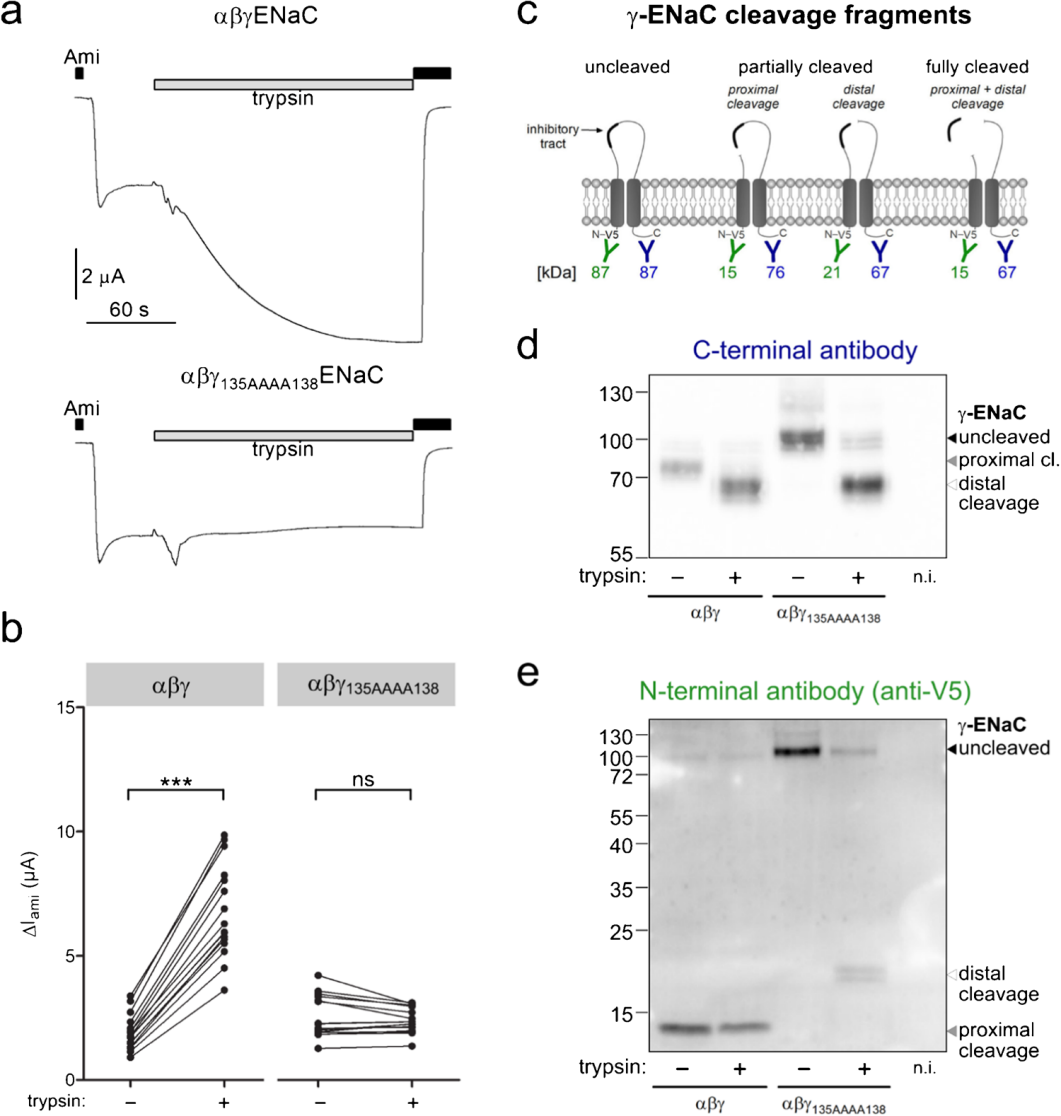
Mutating the proximal cleavage site in γENaC prevents cleavage of this site by endogenous convertases or trypsin. **a** Representative whole-cell current traces recorded using two-electrode voltage-clamp measurements in human wild-type αβγENaC (*top panel*) or mutant αβγ_135AAAA138_ENaC (*bottom panel*) expressing oocytes at a holding potential of − 60 mV. V5-tagged γENaC constructs were used for these experiments to allow parallel western blot experiments shown in Fig. 3 d, e. As indicated by bars, the bath solution contained amiloride (*black bars*; Ami, 2 µM) or trypsin (*grey bars;* trypsin, 2 µg/ml). Note that upon trypsin application an initial transient and variable inward current response was observed with wild-type and mutant ENaC which is probably due to a brief activation of calcium-activated chloride channels [10]. Importantly, a sustained stimulation of Δ*I*_Ami_ by trypsin was only observed with wild-type ENaC but not with the mutant channel which confirmed that the mutation prevented proteolytic channel activation. **b** Summary of absolute Δ*I*_Ami_ values obtained from similar experiments as shown in (**a**). Δ*I*_Ami_ values were obtained before (−) and after application of trypsin (+). Measurements in an individual oocyte are connected by a line. Δ*I*_Ami_ values before and after trypsin application were compared using paired Student’s *t*-test. ***p < 0.001, ns *p* > 0.05. αβγENaC: *n* = 16, *N* = 2, αβγ_135AAAA138_ENaC: *n* = 16, *N* = 2. **c** Schematic diagram showing γENaC cleavage fragments, which can be detected using an antibody raised against a C-terminal γENaC epitope (in blue) or an anti-V5 antibody (in green). The expected molecular weights of the corresponding C- and N-terminal γENaC cleavage fragments are given below in the corresponding color. **d, e** Representative western blots showing cell surface expression of γENaC detected using the C-terminal anti γENaC antibody (**d**) or the N-terminal anti-V5 antibody (**e**) in oocytes expressing wild-type αβγENaC or mutant αβγ_135AAAA138_ENaC either without (−) or with (+) 3-min pretreatment with 2 µg/ml trypsin. Non-injected oocytes served as a control (n.i.). Uncleaved γENaC and cleavage fragments resulting from proximal or distal cleavage are indicated by black, grey, and white arrowheads, respectively. Similar results were obtained in three additional repeats that are shown in Supplementary Figs. 2–4 alongside results from intracellular protein fractions and β-actin staining for blots stained with the anti-V5 antibody, confirming successful separation of intracellular and cell surface protein.

Two-electrode voltage-clamp (TEVC) recordings confirmed previous observations [3, 24, 53, 63] that mutant αβγ_135AAAA138_ENaC is functional with baseline amiloride-sensitive currents similar to wild-type ENaC (2.5 ± 0.2 µA vs. 1.9 ± 0.2 µA; Fig. 3a, b). Importantly, application of trypsin to the bath solution significantly increased ENaC currents only in wild-type but not in αβγ_135AAAA138_ENaC (Fig. 3a, b), indicating that the mutant channel retained the γ-inhibitory peptide in the presence of trypsin due to lack of proximal cleavage.

To validate the cleavage state of γENaC, we performed western blot analysis on cell surface fractions from oocytes expressing wild-type or αβγ_135AAAA138_ENaC (Fig. 3c–e). In wild-type channels, γENaC was predominantly detected as a 76 kDa C-terminal and a 15 kDa N-terminal fragment, corresponding to cleavage at the proximal cleavage site only. Trypsin treatment shifted the C-terminal fragment to 67 kDa, reflecting additional distal cleavage. In contrast, αβγ_135AAAA138_ENaC was detected at ~ 87 kDa with N- or C-terminal antibodies, consistent with uncleaved γENaC. Trypsin treatment of the mutant channel resulted in a 67 kDa C-terminal fragment, confirming distal cleavage. Importantly, the N-terminal fragment was detected at 21 kDa rather than 15 kDa, verifying that the proximal site was still intact after trypsin treatment. These results confirm that the γ_135AAAA138_-mutation prevents endogenous proximal but not trypsin-mediated distal cleavage of γENaC. Together with our functional data, this finding confirms that distal γENaC cleavage is not sufficient to activate ENaC in the absence of proximal cleavage.

**Fig. 4:**
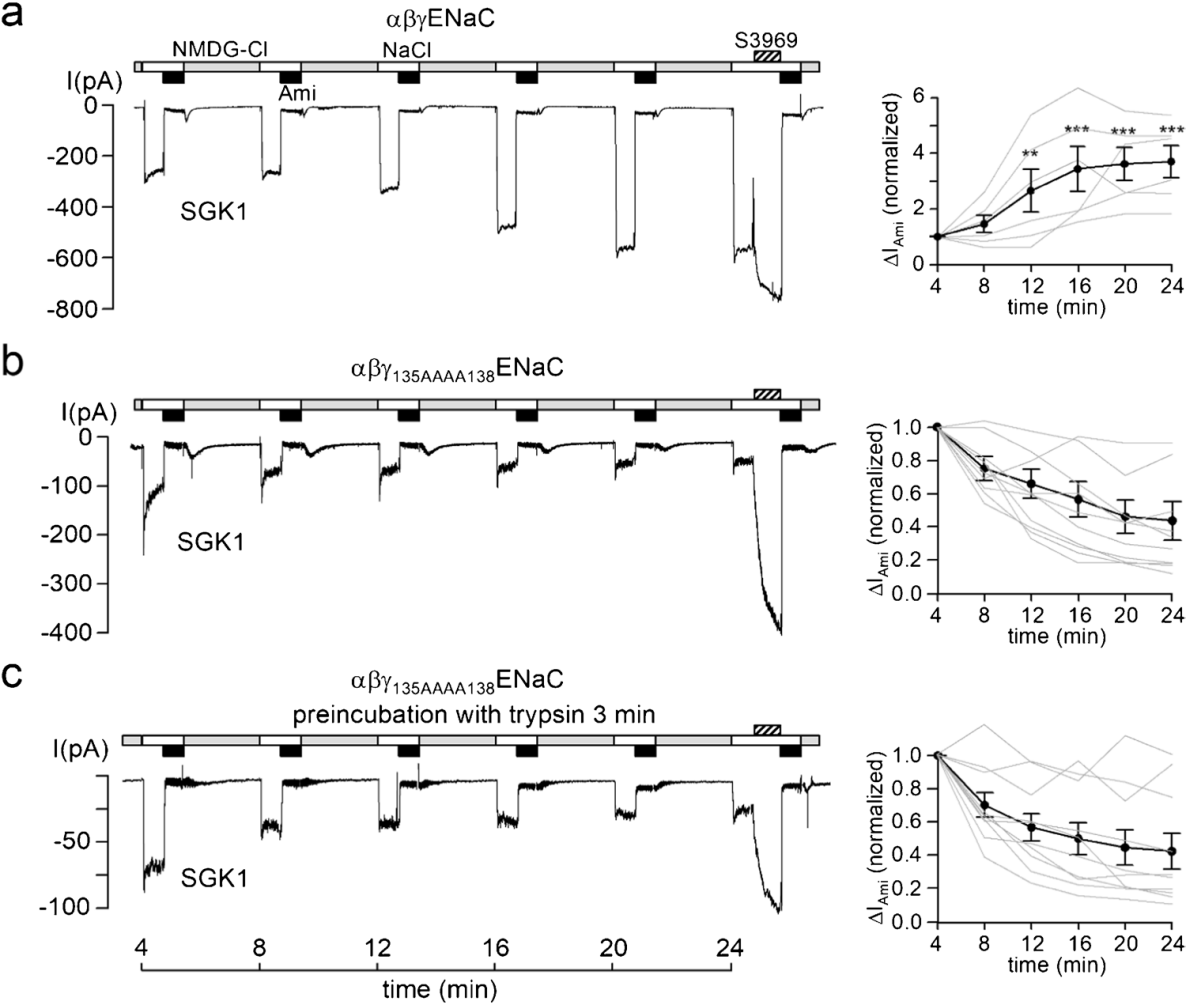
Mutation of the proximal cleavage site in γENaC prevents acute activation of ENaC by SGK1 in outside-out patches. **a, b, c** *Left panels*: Representative current traces recorded in outside-out macropatches of human αβγENaC (**a**) or αβγ_135AAAA138_ENaC (**b**, **c**) as described in Fig. 1. At the end of some experiments (**a** *n* = 2, **b** n = 5, **c** *n* = 4) S3969 (10 µM) was added to the bath solution as indicated by the hatched bars. A quantification of the S3969 effect is provided in Supplementary Fig. 5. Active recombinant SGK1 (SGK1, 80 U/ml) was included in the pipette solution as indicated below the traces. Before patch excision, oocytes were incubated in bath solution without (**a**, **b**) or with 2 µg/ml trypsin (**c**) for 3 min. *Right panels*: Summary of normalized Δ*I*_Ami_ values obtained from similar experiments as shown in the corresponding left panels using the same symbols as in Fig. 1. Δ*I*_Ami_ values at individual time points were compared with the corresponding initial Δ*I*_Ami_ value at 4 min using repeated-measures ANOVA with Dunnett's post-hoc test of log-transformed values. No statistical testing was performed in absence of stimulation by SGK1 (**b**, **c**). **p < 0.01, ***p < 0.001. **a** *n* = 6, *N* = 4, **b** *n* = 10, *N* = 7, **c** *n* = 10, *N* = 6

We next tested whether SGK1 could stimulate αβγ_135AAAA138_ENaC in outside-out macropatch recordings (Fig. 4). Initial ENaC currents in patches with the mutant channel were similar to those from patches with wild-type ENaC, consistent with our TEVC findings. Importantly, SGK1 produced its typical stimulatory effect on ENaC currents only in patches with wild-type ENaC (Fig. 4a) but not in patches with αβγ_135AAAA138_ENaC (Fig. 4b). Interestingly, when the stimulatory effect of SGK1 had reached a plateau in patches with wild-type ENaC, application of the small molecule ENaC activator S3969 resulted only in a modest additional current increase of about 40% (Fig. 4a, Supplementary Fig. 5). In the concentration used (10 µM), S3969 is thought to increase ENaC activity to its maximal level, i.e., an open probability of ~ 1 [41, 64]. Thus, the rather small additional effect of S3969 indicates that SGK1 almost fully stimulates the activity of wild-type ENaC. In contrast, in patches with αβγ_135AAAA138_ENaC and SGK1 in the pipette solution, application of S3969 at the end of the experiment resulted in a large ~ 15-fold current increase (Fig. 4b, Supplementary Fig. 5). This indicates that the baseline open probability of αβγ_135AAAA138_ENaC is rather low but can be increased substantially by S3969. Thus, the failure of SGK1 to activate ENaC currents in patches with αβγ_135AAAA138_ENaC was not due to a general inability of these channels to increase their activity but to a lack of responsiveness to SGK1. Finally, we tested whether SGK1 could stimulate αβγ_135AAAA138_ENaC after trypsin treatment in a condition where γENaC is cleaved distally but not proximally. However, even with this distal cleavage, SGK1 had no stimulatory effect on αβγ_135AAAA138_ENaC currents, while the current increase observed with S3969 was still high (approx. sevenfold) (Fig. 4c, Supplementary Fig. 5).

In summary, these experiments demonstrate that SGK1-mediated ENaC activation requires prior proteolytic γENaC cleavage at its proximal cleavage site.

#### Intramolecular linkage resulting in covalent binding of the γ-inhibitory peptide to its binding site prevents acute ENaC activation by SGK1

Our previous experiments demonstrated that SGK1 effectively stimulates ENaC when the channel’s γ-subunit is cleaved at its proximal cleavage site (Fig. 4a), but not when it is fully cleaved or cleaved at its distal but not its proximal site (Fig. 4c). Cleavage only at the proximal site does not result in the complete release of the γ-inhibitory tract but is likely to permit some movement of the γ-inhibitory tract away from its binding site. This increased mobility of the γ-inhibitory tract due to proximal cleavage may be a prerequisite for channel activation by SGK1.

**Fig. 5:**
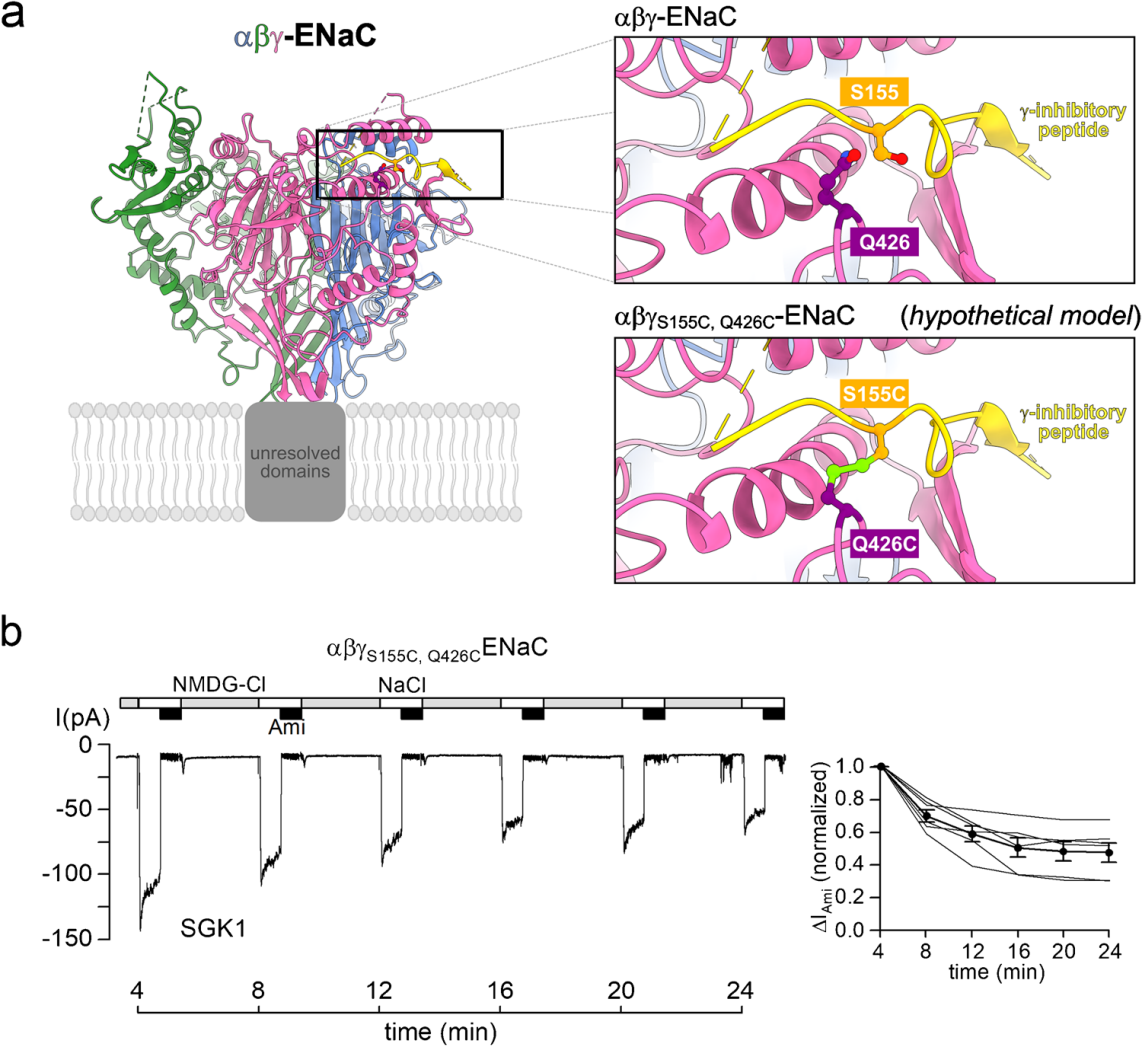
Covalent binding of the γ-inhibitory peptide to its binding site by introducing a disulfide bond prevents activation of ENaC by SGK1.** a** A ribbon diagram of the extracellular loop of human αβγENaC generated using atom coordinates from PDB entry 6WTH [47]. The α-subunit is colored in blue, β-subunit in dark green, and γENaC in pink with its inhibitory peptide in yellow. The putative location of the unresolved transmembrane domains is indicated with a grey box placed within a schematically depicted lipid bilayer. The top inset shows the γ-inhibitory peptide within its binding site on a larger scale. Serine residue 155 and glutamine residue 426 are shown in a ball and stick representation with oxygens in red, nitrogens in blue, and carbons in orange for Ser155 and violet for Gln426, respectively. Hydrogen atoms are omitted for clarity. The bottom inset shows a model of αβγ_S155C,Q426C_ENaC based on the cryo-EM data for wild-type ENaC where S155 and Q426 were mutated to cysteine residues in silico. Sulfur atoms and the newly formed disulfide bond are shown in light green. This visualisation and the *in silico* mutagenesis were prepared using UCSF Chimera developed by the Resource for Biocomputing, Visualization, and Informatics at the University of California, San Francisco [50]. **b** *Left panel*: Representative current traces recorded in an outside-out macropatch of an αβγ_S155C,Q426C_ENaC expressing oocyte, as described in Fig. 1. Active recombinant SGK1 (SGK1, 80 U/ml) was included in the pipette solution as indicated below the trace. *Right panel*: Summary of normalized Δ*I*_Ami_ values obtained from similar experiments as shown in the left panel using the same symbols as in Fig. 1. *n* = 6, *N* = 5

To test this hypothesis, we examined a γENaC mutant with a γ-inhibitory peptide that is covalently locked to its binding site. As shown previously [63], introducing cysteine substitutions at Ser155 (within the γ-inhibitory peptide) and Gln426 (in its binding pocket) in γENaC (γ_S155C,Q426C_) results in spontaneous disulfide bond formation, effectively tethering the inhibitory peptide to its binding site without affecting proteolytic cleavage per se. Notably, this disulfide bond is located closer to the proximal end of the inhibitory peptide and is likely to restrict the movement of the γ-inhibitory peptide away from its binding site even when the proximal site is cleaved (Fig. 5a).

Interestingly, SGK1 failed to increase ENaC-mediated currents in patches with αβγ_S155C,Q426C_ENaC (Fig. 5b). This suggests that SGK1-mediated ENaC activation requires a certain degree of mobility for the γ-inhibitory peptide, which is normally achieved by proximal cleavage but is absent in mutant αβγ_S155C,Q426C_ENaC.

## Discussion

In this study, we made the following key observations: (i) in outside-out patches SGK1 acutely stimulates human ENaC activity in a similar manner as previously shown for rat ENaC; (ii) this stimulation critically depends on the conserved serine residue S594 in the intracellular C-terminus of human αENaC; (iii) the stimulatory effect of SGK1 is not additive to proteolytic ENaC activation; (iv) preventing proteolytic cleavage at the proximal cleavage site in γENaC abolishes the stimulatory SGK1 effect; and (v) covalently tethering the γ-inhibitory peptide to its binding site via an engineered disulfide bond impedes SGK1-mediated ENaC stimulation. Together, these results reveal that acute SGK1-mediated stimulation of human ENaC is critically dependent on S594 in αENaC and intricately linked to proteolytic processing of the channel’s γ-subunit, emphasizing a previously underappreciated interplay between kinase and protease regulatory pathways.

It is generally accepted that a sustained stimulation of ENaC activity by SGK1 involves enhanced channel expression at the cell surface as a key mechanism. This occurs through enhanced channel forward trafficking to the plasma membrane [1, 37, 38] and reduced Nedd4-2-mediated ubiquitylation and subsequent channel internalization [1, 8, 12, 13, 15, 34, 52, 57, 59–61]. In addition, we demonstrated that SGK1 acutely increased ENaC-mediated currents by about twofold in outside-out macropatches from *Xenopus laevis* oocytes expressing rat ENaC [10, 11, 33]. Most likely, this stimulatory effect was not caused by an insertion of additional channels into the patch but was strictly dependent on a critical phosphorylation site in rat αENaC (S621). Presently, it is still unclear whether SGK1 directly phosphorylates this site or mediates its phosphorylation by activating other kinases, like DYRK2 [11], by inhibiting phosphatases [10] or by inhibiting inhibitory kinases, like, e.g., glycogen synthase kinase-3 beta (GSK3β) [11]. In any case, our previous findings suggested that in the presence of SGK1, phosphorylation of S621 in the channel’s α-subunit acutely stimulated rat ENaC activity. Recordings with single channel resolution suggested that this was mainly achieved by increasing the open probability of so-called near-silent channels. This latter channel population is characterized by a baseline open probability below ~ 0.05 [4, 5, 9, 14, 22, 29]. In the current study, we confirmed that SGK1 also acutely increased human ENaC currents in excised outside-out patches. In line with previous studies [10–12, 33], the onset of SGK1-dependent ENaC stimulation was observed approximately 4–8 min after patch excision. This latency could reflect the involvement of other kinases and phosphatases and/or a technical delay caused by bath solution drawn into the pipette tip during patch excision which is likely to dilute SGK1 and slow down its diffusion to the membrane patch. Like with the rat orthologue [10], the acute stimulatory effect of SGK1 critically depended on a homologous serine residue (S594) in the intracellular C-terminus of human αENaC. In contrast, in whole-cell recordings, SGK1 coexpression still enhanced ENaC currents when this site was mutated in rat αENaC [12, 16] or deleted by a premature stop codon in human αENaC [67]. Taken together, these observations support the concept that SGK1 stimulates ENaC activity by two distinct mechanisms. Firstly, SGK1 promotes ENaC expression at the cell surface probably by inhibiting channel retrieval and by enhancing forward trafficking of the channel to the plasma membrane. Secondly, SGK1 can acutely increase the channel’s open probability. Only this latter effect is dependent on the S594 phosphorylation site as demonstrated in our outside-out patch clamp recordings. This acute effect may represent a physiologically relevant rapid initial adaptation of channel activity and is likely to precede the effect of SGK1 to increase channel expression at the cell surface required for long-term adaptation of channel activity.

Contrary to our findings with αβγENaC, SGK1 failed to stimulate δβγENaC activity in outside-out patches. This lack of an acute stimulatory effect of SGK1 on δβγENaC may be explained by the previously reported finding [21] that the basal open probability of δβγENaC was ~ 1 in excised patches, preventing any further increase. In contrast, in coexpression studies, the neuronal SGK1 isoform SGK1.1 has been reported to increase δβγENaC currents by enhancing channel expression at the cell surface [70]. This highlights the need to distinguish between acute and long-term stimulatory effects of SGK1 on ENaC.

This study focused on the interaction of protease- and kinase-mediated activation of αβγENaC. In *Xenopus laevis* oocytes, αENaC is fully proteolytically processed at the cell surface, while only the proximal cleavage site of γENaC is processed intracellularly [3, 9, 20, 24, 25]. The precise physiological role of this initial proximal cleavage of γENaC is still unclear. Preventing this proximal cleavage by mutating the corresponding γENaC cleavage site did not alter baseline ENaC currents, a finding consistent with earlier reports [3, 24, 53, 63]. Therefore, we expected SGK1 to stimulate this mutant channel in a similar manner as wild-type ENaC. Surprisingly, SGK1 no longer stimulated amiloride-sensitive currents in αβγ_135AAAA138_ENaC with abolished proximal γENaC cleavage. Moreover, when only the distal cleavage site in γENaC was cleaved by incubating αβγ_135AAAA138_ENaC expressing oocytes in trypsin, SGK1 did not lead to an increase in ENaC activity. Taken together, these results indicate that proximal cleavage of γENaC is a prerequisite for SGK1-mediated ENaC stimulation. Thus, our findings suggest a functional link between kinase- and protease-mediated ENaC activation. Recent work by Ray et al. using a similar mutation in mice reported no overt phenotype under baseline conditions or when challenged with restricted sodium or increased potassium intake [53]. One possible explanation is that impaired γENaC cleavage expected to prevent normal proteolytic channel activation and acute ENaC stimulation by SGK1 may be compensated by increased ENaC surface expression or by compensatory mechanisms in other tubular segments. Additional studies are needed to explore a possible interdependence of proteolytic ENaC activation and the effect of SGK1 on channel trafficking.

In our experiments, we observed no further stimulatory effect of SGK1 on ENaC-mediated currents when ENaC was proteolytically activated by incubating oocytes in trypsin. Within the *Xenopus laevis* oocyte expression system, trypsin increases the open probability of ENaC close to ~ 1 [4, 9, 20]. Under these conditions, the total current solely depends on the number of channels in the patch. Therefore, the lack of additional ENaC stimulation by SGK1 suggests that the acute effect of SGK1 in outside-out patches on membrane trafficking is negligible. Thus, the acute SGK1 stimulation observed in the absence of proteolytic pre-activation is best explained by an increase in ENaC’s open probability. In contrast to the *Xenopus laevis* expression system, where ENaC open probability approaches unity after complete proteolytic ENaC activation, the open probability of fully cleaved ENaC may only reach ~ 0.3 in native epithelial cells [17, 18, 44, 45]. Therefore, it is conceivable that *in* *vivo* SGK1 may further increase the open probability of fully cleaved ENaC unlike in the oocyte expression system.

It is tempting to speculate about conformational changes that occur following phosphorylation of αENaC at S621 or S594 in rat or human, respectively. Unfortunately, high-resolution structures of ENaC transmembrane helices and the adjacent intracellular segments are not yet available. AlphaFold modeling of human αENaC (AlphaFold ID: P37088) places S594 immediately C-terminal to the second transmembrane helix [27, 66], albeit with a low confidence score. Introducing a negatively charged phosphate at this position may alter local interactions between the helix and the inner leaflet of the plasma membrane, potentially affecting channel gating. From our study, the question arises which molecular mechanism may mediate the apparent functional link between the conserved phosphorylation site in the cytosolic C-terminus of αENaC and the extracellularly located binding site of the γ-inhibitory peptide [46, 47]. As the increase in ENaC current triggered by phosphorylation at this site is reminiscent of that observed with proteolytic channel activation, a possible molecular explanation could be that phosphorylation in αENaC induces conformational changes that ultimately favor the dissociation of the γ-inhibitory peptide from its binding site. Our observation that covalent binding of the γ-inhibitory peptide to its binding site in αβγ_S155C,Q426C_ENaC prevents the SGK1-mediated stimulation supports this hypothesis. Interestingly, the binding site of the γ-inhibitory peptide is formed by a central α-helix that serves on its opposite side as a molecular interface to the adjacent αENaC subunit [46, 47]. It seems plausible that through this molecular interface conformational changes in αENaC can affect the binding site of the inhibitory peptide in γENaC favoring the dissociation of the inhibitory peptide. Alternatively, a release of the γENaC inhibitory peptide may enhance the channel’s conformational flexibility, enabling αENaC phosphorylation to be transduced into gating changes. Obviously, these considerations regarding potential molecular mechanisms linking proteolytic channel activation and channel activation by phosphorylation remain speculative due to the limited availability of high-resolution structural data for ENaC, in particular of its cytosolic and transmembrane domains and of the channel’s open state. Thus, a further analysis of the channel structure is needed to elucidate the underlying molecular mechanisms of proteolytic ENaC activation and its interplay with channel activation by kinases.

In summary, our study provides evidence that an acute SGK1-mediated activation of human ENaC is critically dependent on a conserved serine residue (S594) in the channel’s α-subunit and requires proteolytic processing of the γ-subunit at its proximal cleavage site. Thus, hormonal and proteolytic pathways of ENaC regulation appear to be linked, which may be (patho-)physiologically relevant in fine-tuning ENaC activity in health and disease. 

## Supplementary Information

Below is the link to the electronic supplementary material.

Supplementary file1 (PDF 818 KB)

Supplementary file1 (PDF 506 KB)

## Data Availability

All data is included in the manuscript and the supplementary information.
