# Supplementary material for: Acute activation of human epithelial sodium channel (ENaC) by serum and glucocorticoid inducible kinase 1 (SGK1) requires prior cleavage of the channel’s γ-subunit at its proximal cleavage site
Source: Pflugers Arch. 2025 Jun 21;477(8):1061–74. doi: 10.1007/s00424-025-03099-z (PMC12310850; doi:10.1007/s00424-025-03099-z)
Supplement: Supplementary file 1 — Supplementary file1 (PDF 818 KB) [file 424_2025_3099_MOESM1_ESM.pdf]

Acute activation of human epithelial sodium channel (ENaC) by serum and glucocorticoid inducible kinase 1 (SGK1) requires prior cleavage of the channel's γ-subunit at its proximal cleavage site

Alexei Diakov, Florian Sure, Alexandr V. Ilyaskin and Christoph Korbmacher

Friedrich-Alexander-Universität Erlangen-Nürnberg, Institute of Cellular and Molecular Physiology, Erlangen, Germany

Supplementary Information – Table of contents

Supplementary Figure 1:	Recombinant SGK1 fails to stimulate δβγENaC currents in outside-out patches
Supplementary Figure 2:	Western blot detection of C-terminal cleavage fragments of γENaC
Supplementary Figure 3:	Western blot detection of N-terminal cleavage fragments of γENaC
Supplementary Figure 4:	Confirmation of separation between cell surface and intracellular proteins using β-actin staining
Supplementary Figure 5:	Quantification of the effect of S3969 on ΔI_{Ami} at the plateau of the SGK1 effect

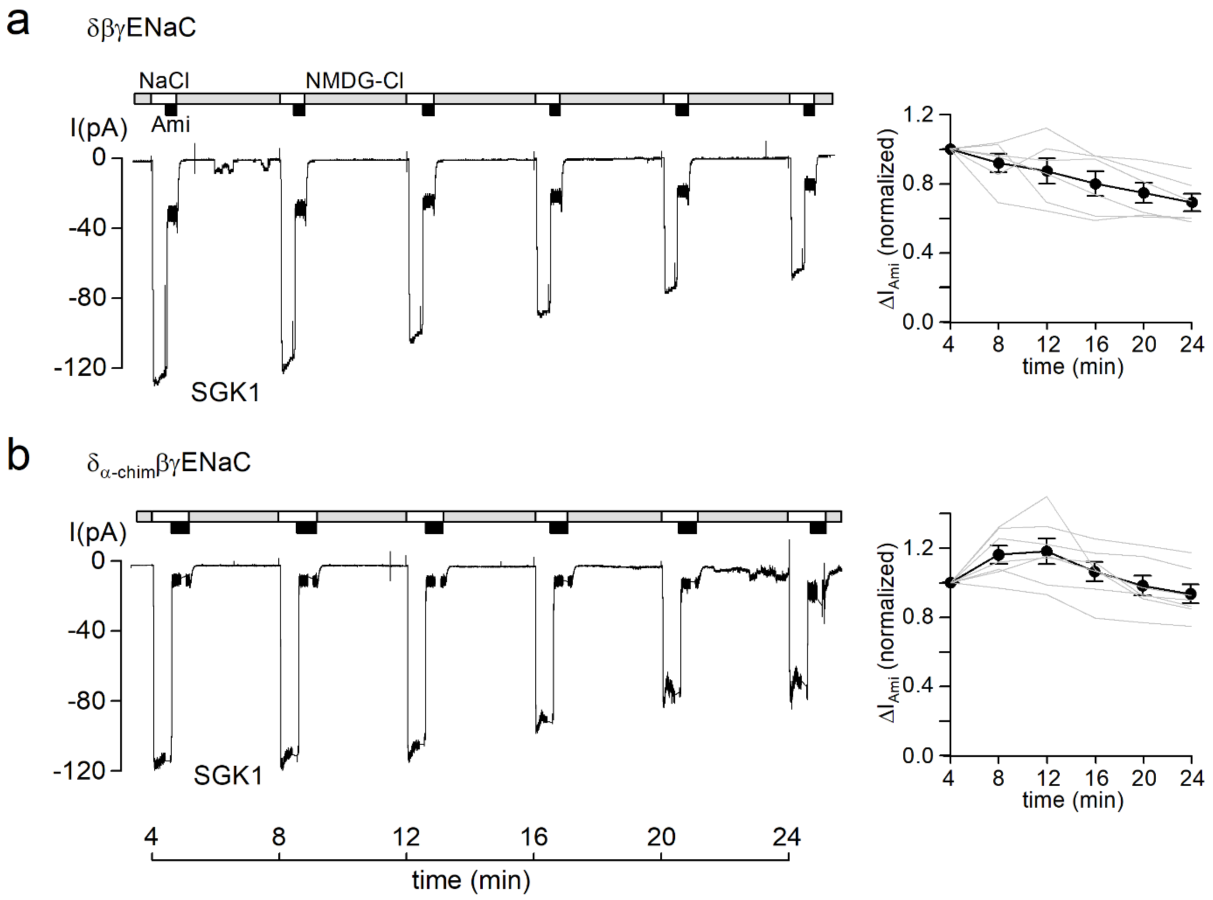

Supplementary Fig. 1 Recombinant SGK1 fails to stimulate δβγENaC currents in outside-out patches

(a, b) *Left panels*: Representative current traces recorded in outside-out macropatches from an oocyte expressing human δβγENaC (a) or ENaC with wild-type β- and γ-subunits and a chimeric δ-α-subunit ($\delta_{\alpha\text{-chim}}$; residues 1-538 from δENaC, residues 562-669 from αENaC). The same experimental protocol was used as described in Fig. 1, but with a higher concentration of amiloride (100 µM). Active recombinant SGK1 (SGK1; 80 U/ml) was included in the pipette solution as indicated below the traces. *Right panels*: Summary of normalized ΔI_{Ami} values obtained from similar experiments as shown in the corresponding left panels using the same symbols as in Fig. 1. a: n=6, N=4, b: n=7, N=5.

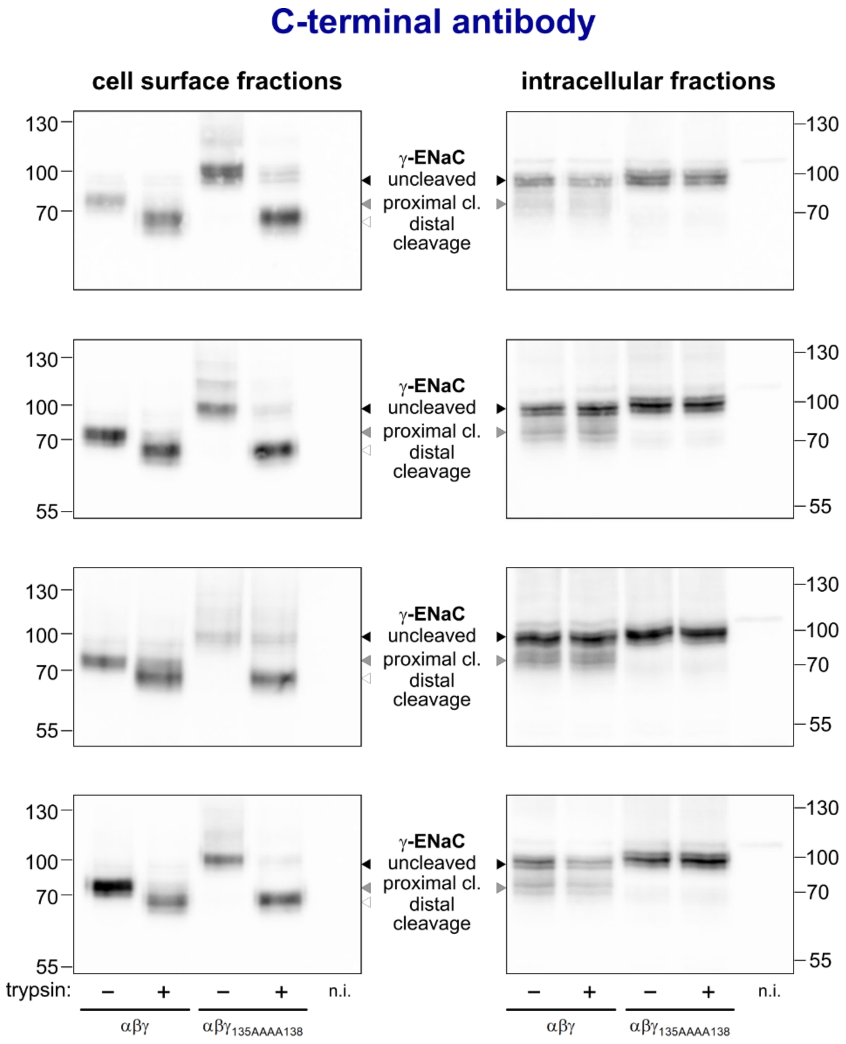

Supplementary Fig. 2 Western blot detection of C-terminal cleavage fragments of γENaC
Western blots showing cell surface (left panels) and corresponding intracellular (right panels) expression of γENaC detected using the C-terminal anti γENaC antibody in four batches of oocytes expressing wildtype αβγENaC or mutant $\alpha\beta\gamma_{135AAAA138}$ENaC either without (−) or with (+) 3 min pretreatment with 2 µg/ml trypsin as indicated. Noninjected oocytes served as control (n.i.). Uncleaved γENaC and cleavage fragments resulting from proximal or distal cleavage are indicated by black, grey and white arrowheads, respectively. The blot in the top left panel is the same as shown in Figure 3d.

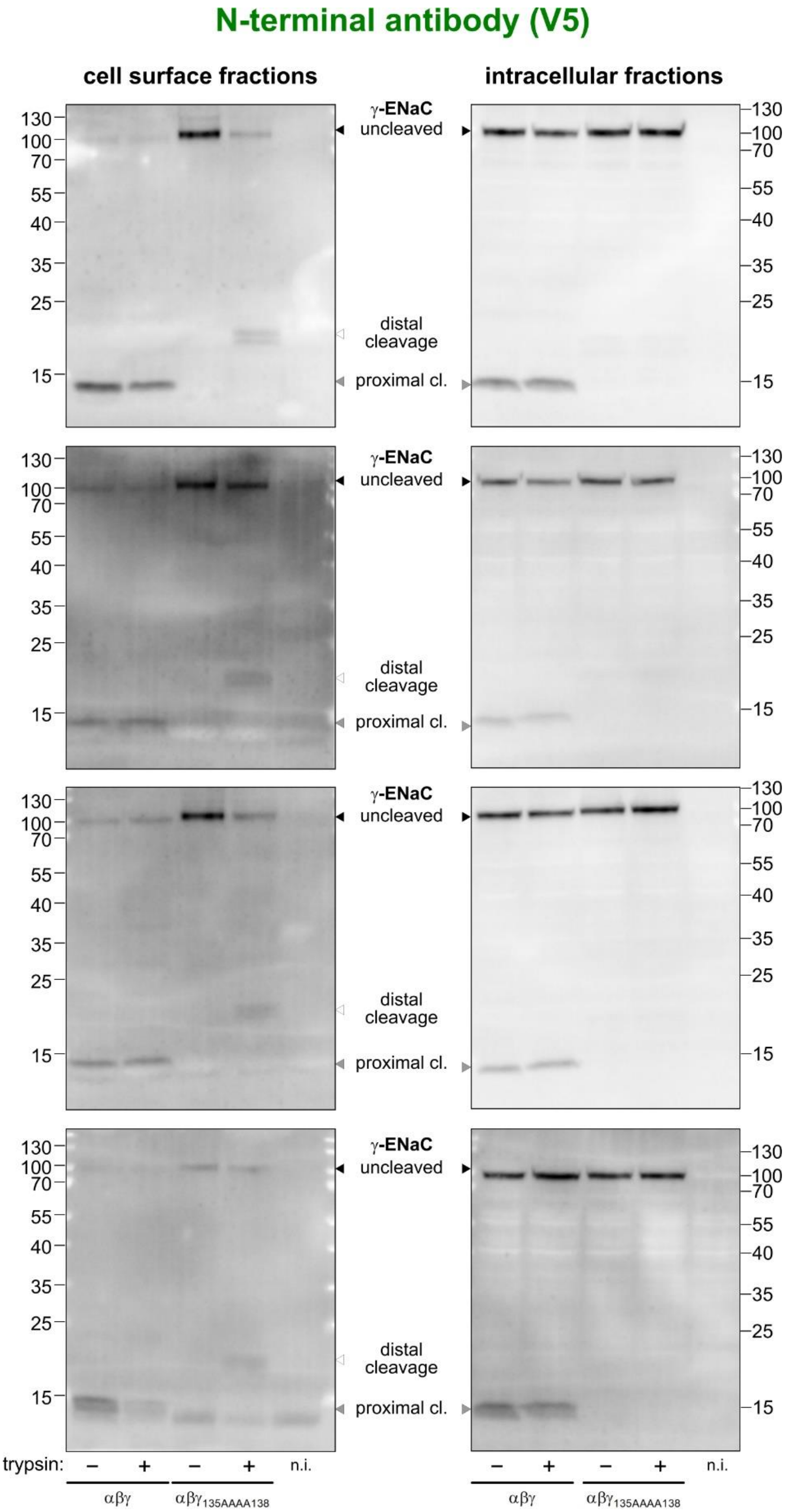

Supplementary Fig. 3 Western blot detection of N-terminal cleavage fragments of γENaC
Western blots showing cell surface (left panels) and corresponding intracellular (right panels) expression of γENaC detected using the N-terminal anti-V5 antibody in four batches of oocytes expressing wildtype αβγENaC or mutant $\alpha\beta\gamma_{135AAAA138}$ENaC either without (−) or with (+) 3 min pretreatment with 2 µg/ml trypsin. Noninjected oocytes served as a control (n.i.). Uncleaved γENaC and cleavage fragments resulting from proximal or distal cleavage are indicated by black, grey and white arrowheads, respectively. The blot in the top left panel is the same as shown in Figure 3e.

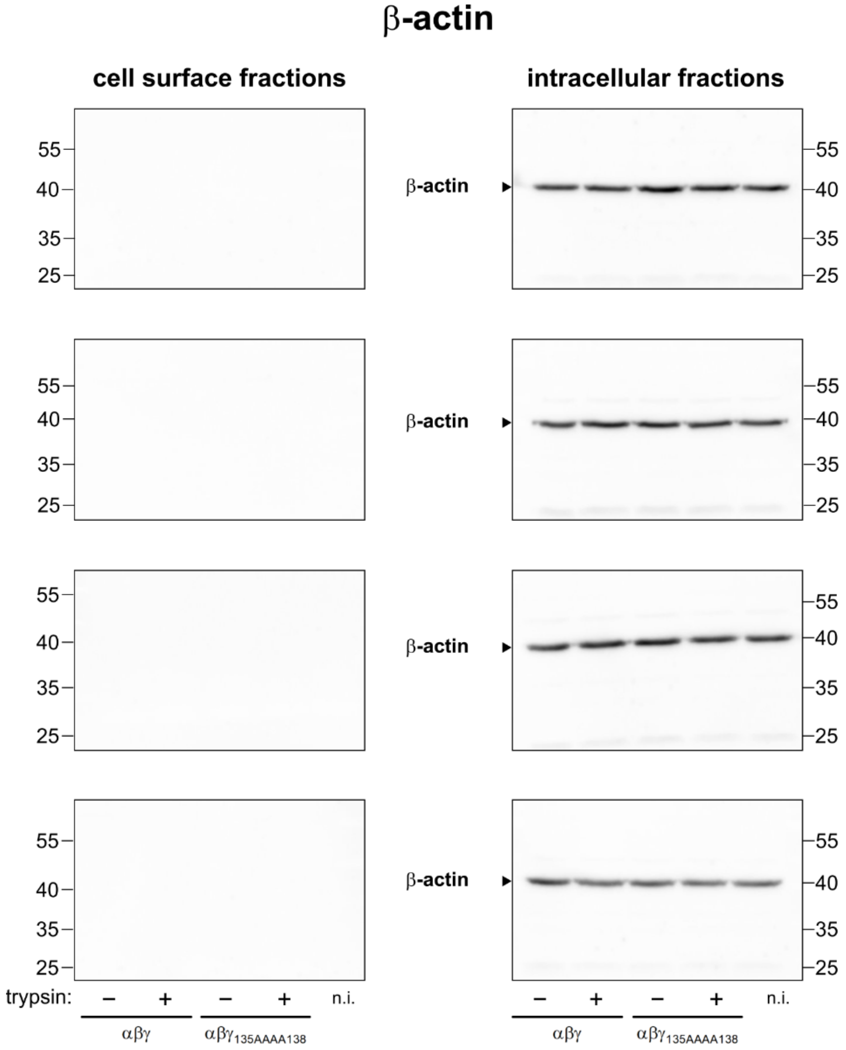

Supplementary Fig. 4 Confirmation of separation between cell surface and intracellular proteins using β-actin staining

Western blots showing cell surface (left panels) and corresponding intracellular (right panels) expression of β-actin (blots shown in Supplementary Fig. 3 were stripped and reprobed with an anti-β-actin antiserum) in four batches of oocytes expressing wildtype αβγENaC or mutant $\alpha\beta\gamma_{135AAAA138}$ENaC either without (−) or with (+) 3 min pretreatment with 2 µg/ml trypsin. Noninjected oocytes served as a control (n.i.). The absence of a signal in the cell surface fraction confirms successful separation of cell surface proteins.

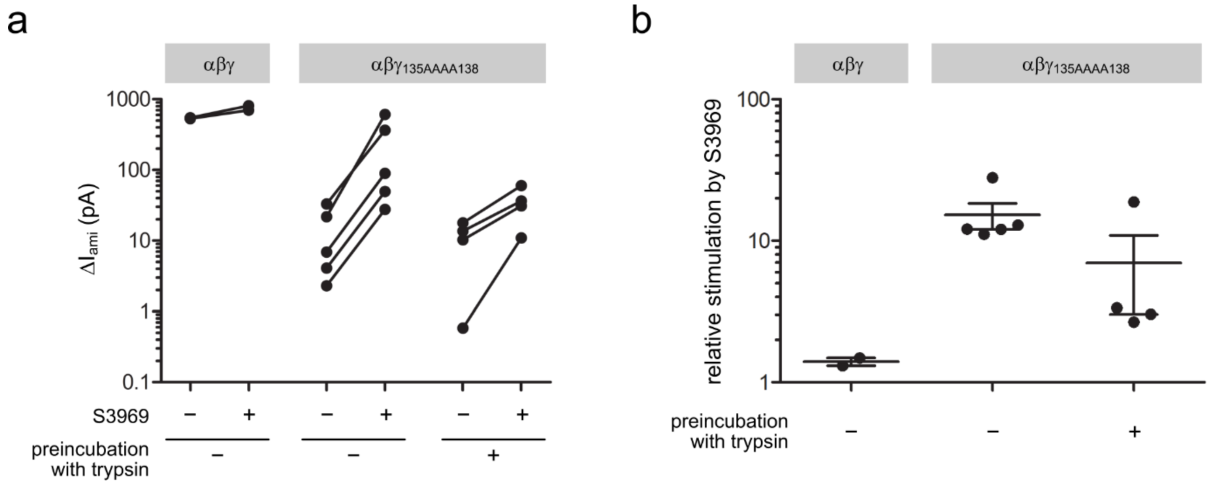

Supplementary Fig. 5 Quantification of the effect of S3969 on ΔI_{Ami} at the plateau of the SGK1 effect

(a) Summary of absolute ΔI_{Ami} values obtained from similar experiments as shown in Figure 3 in oocytes expressing αβγENaC or $\alpha\beta\gamma_{135AAAA138}$ENaC without or with preincubation for 3 min in trypsin, as indicated. Values at the timepoint t=24 min when the effect of SGK1 had reached a plateau immediately before (−) and after the addition (+) of S3969 are shown on a logarithmic scale. **(b)** The relative stimulatory of S3969 on ΔI_{Ami} was calculated from the data shown in (a) by dividing the ΔI_{Ami} reached in presence of S3969 by the value reached before the addition of S3969. Mean ± SEM and individual datapoints are shown on a logarithmic scale. αβγENaC: n=2, N=1, $\alpha\beta\gamma_{135AAAA138}$ without trypsin preincubation: n=5, N=3, $\alpha\beta\gamma_{135AAAA138}$ with trypsin preincubation: n=4, N=3.
