# Supplementary material for: Acute activation of human epithelial sodium channel (ENaC) by serum and glucocorticoid inducible kinase 1 (SGK1) requires prior cleavage of the channel’s γ-subunit at its proximal cleavage site
Source: Pflugers Arch. 2025 Jun 21;477(8):1061–74. doi: 10.1007/s00424-025-03099-z (PMC12310850; doi:10.1007/s00424-025-03099-z)

Acute activation of human epithelial sodium channel (ENaC) by serum and glucocorticoid inducible kinase 1 (SGK1) requires prior cleavage of the channel's γ-subunit at its proximal cleavage site

Alexei Diakov, Florian Sure, Alexandr V. Ilyaskin and Christoph Korbmacher

Friedrich-Alexander-Universität Erlangen-Nürnberg, Institute of Cellular and Molecular Physiology, Erlangen, Germany

Full, uncropped western blots

Lane order

Lane	ENaC	Trypsin
**1**	αβγ	–
**2**	αβγ	+
**3**	$\alpha\beta\gamma_{135AAAA138}$	–
**4**	$\alpha\beta\gamma_{135AAAA138}$	+
**5**	non-injected	–

C-terminal antibody

cell surface fractions / intracellular fractions

Figure 3d and Supplemental Figure 2, top row, left panel

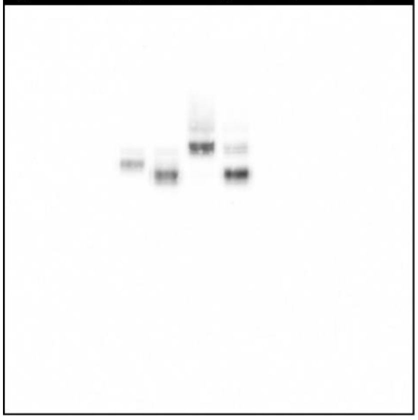

Supplemental Figure 2, top row, right panel

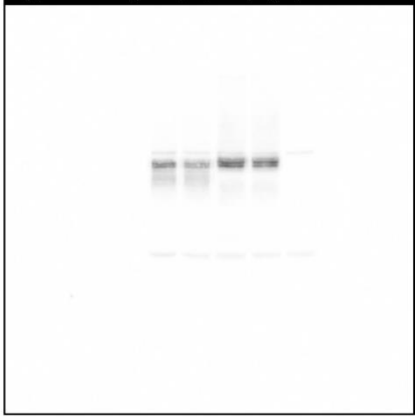

Supplemental Figure 2, second row, left panel

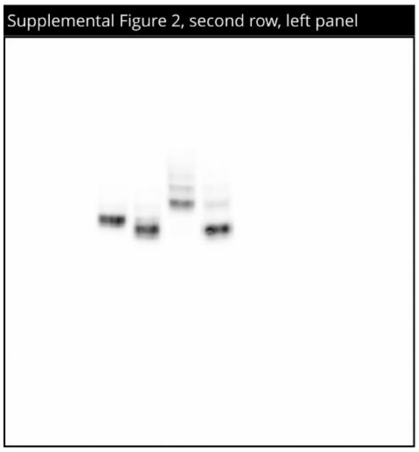

Supplemental Figure 2, second row, right panel

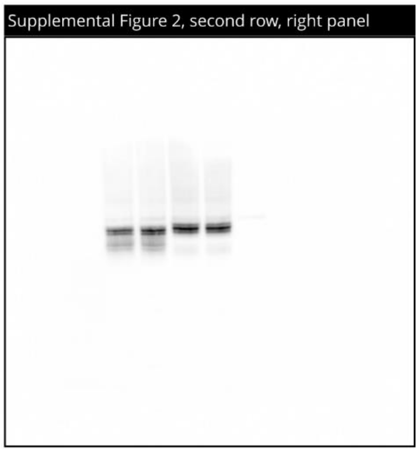

Supplemental Figure 2, third row, left panel

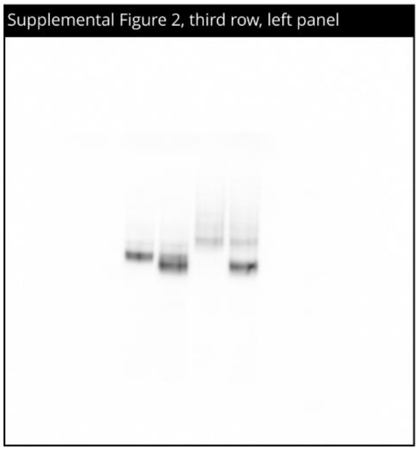

Supplemental Figure 2, third row, right panel

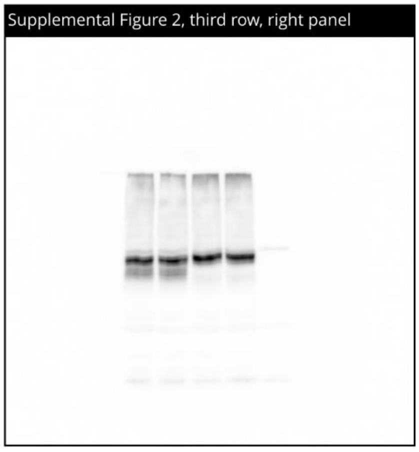

Supplemental Figure 2, bottom row, left panel

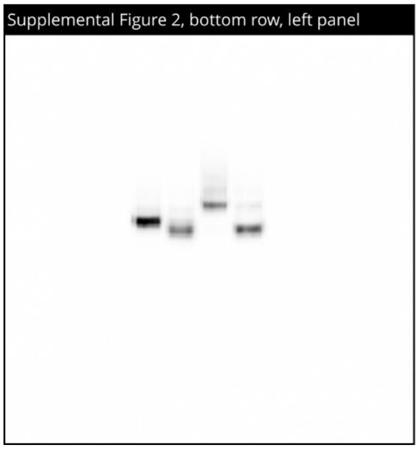

Supplemental Figure 2, bottom row, right panel

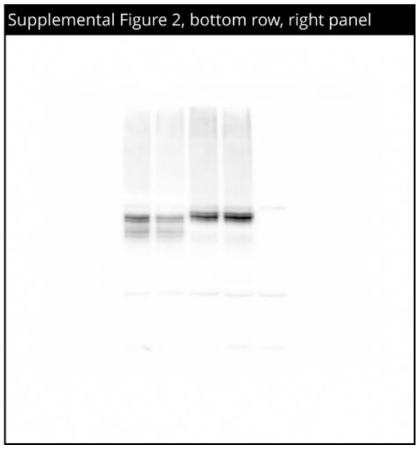

N-terminal antibody (V5)

cell surface fractions

intracellular fractions

Figure 3e and Supplemental Figure 3, top row, left panel

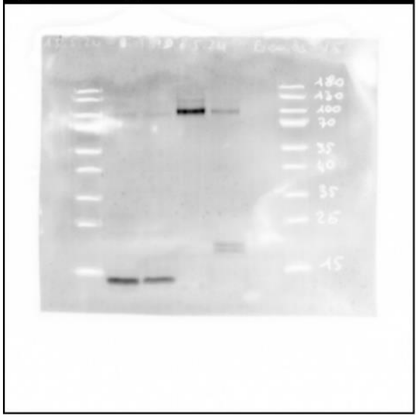

Supplemental Figure 3, top row, right panel

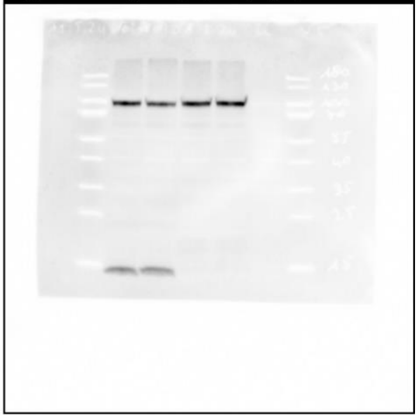

Supplemental Figure 3, second row, left panel

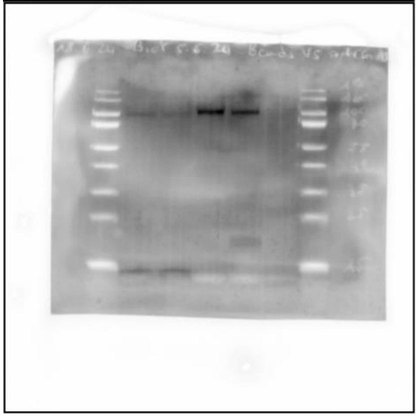

Supplemental Figure 3, second row, right panel

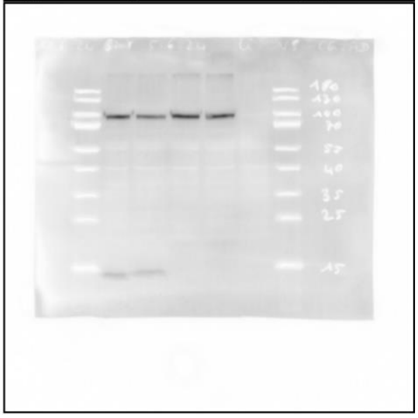

Supplemental Figure 3, third row, left panel

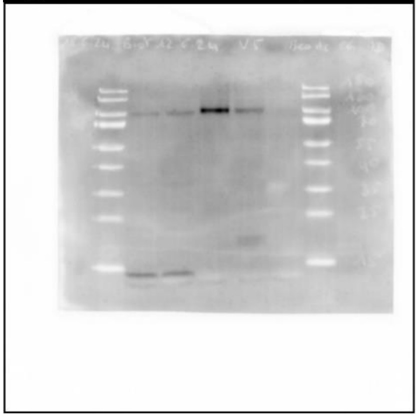

Supplemental Figure 3, third row, right panel

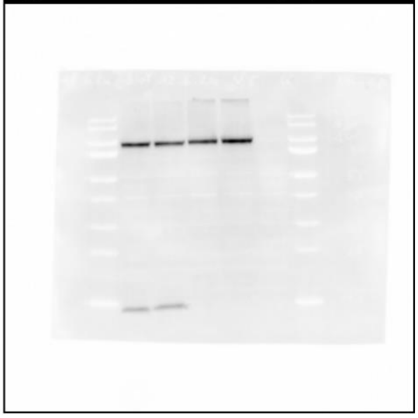

Supplemental Figure 3, bottom row, left panel

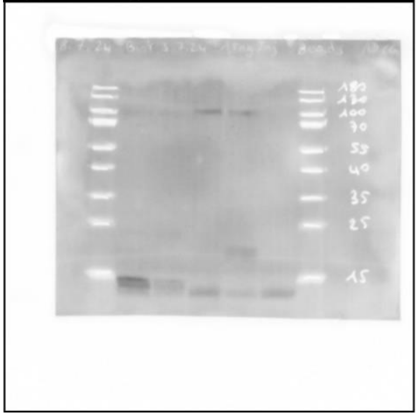

Supplemental Figure 3, bottom row, right panel

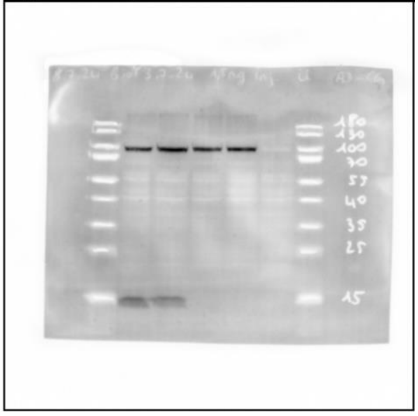

β-actin

cell surface fractions | **intracellular fractions**

Supplemental Figure 4, top row, left panel

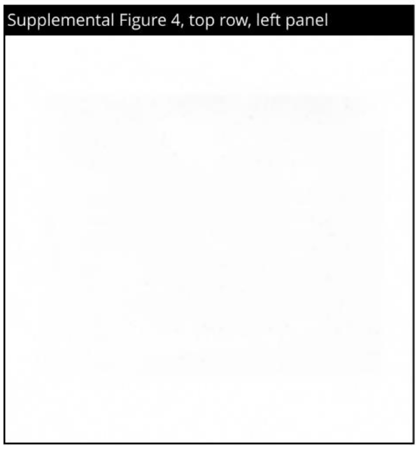

Supplemental Figure 4, top row, right panel

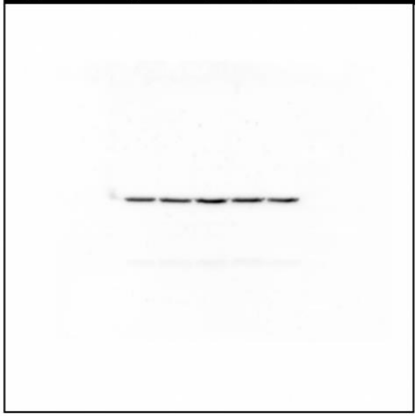

Supplemental Figure 4, second row, left panel

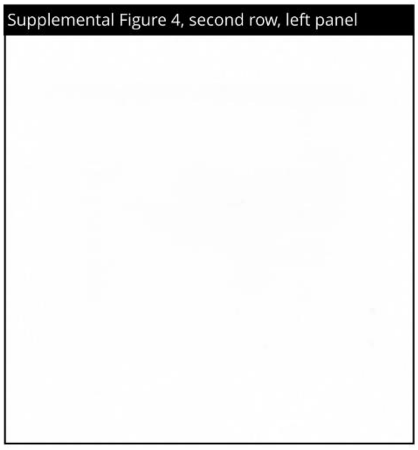

Supplemental Figure 4, second row, right panel

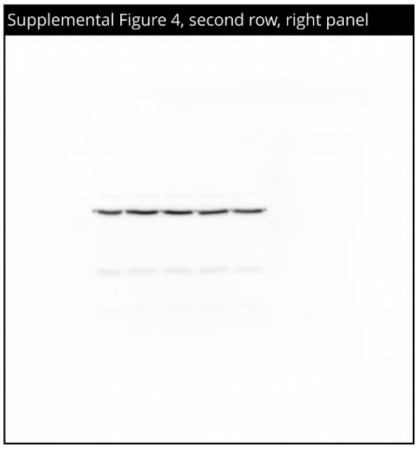

Supplemental Figure 4, third row, left panel

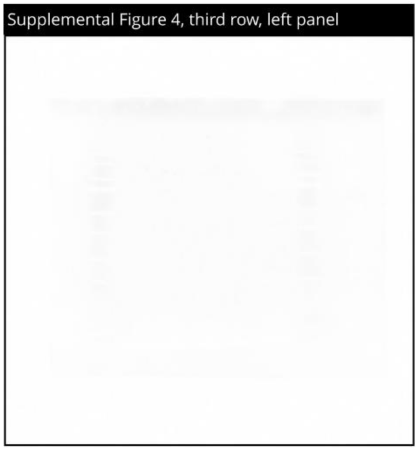

Supplemental Figure 4, third row, right panel

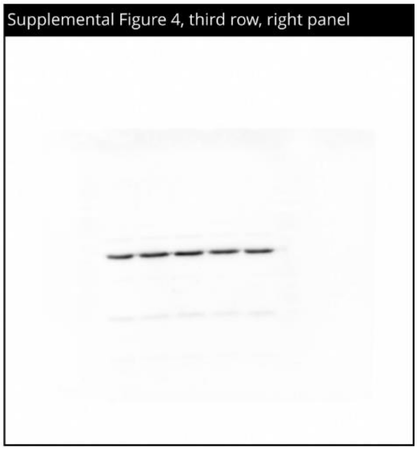

Supplemental Figure 4, bottom row, left panel

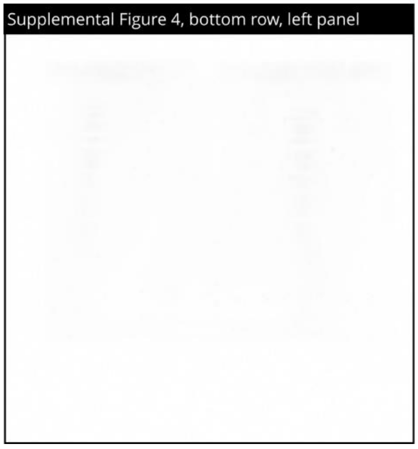

Supplemental Figure 4, bottom row, right panel

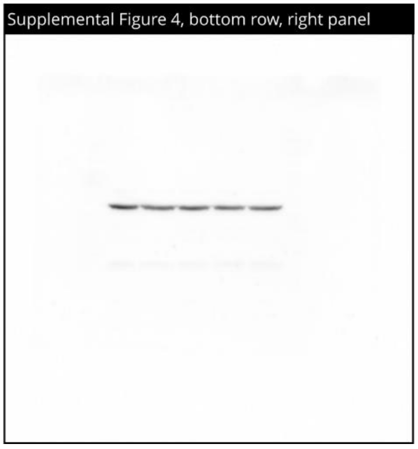

Supplement: Supplementary file 2 — Supplementary file1 (PDF 506 KB) [file 424_2025_3099_MOESM2_ESM.pdf]
